# Lecanemab Real‐World Use Perspectives from a New England Alzheimer’s Disease Infusion Center: A Retrospective Chart Review

**DOI:** 10.1002/alz70859_101388

**Published:** 2025-12-25

**Authors:** Salvatore Napoli, Rachel O'Donnell, Lauren Nassr, Emily Maize

**Affiliations:** ^1^ Alzheimer’s Neurology and Infusion Centers of New England, Foxborough, MA USA

## Abstract

**Background:**

Lecanemab is a monoclonal antibody targeting neurotoxic Aβ protofibrils and plaques, which has been shown to significantly slow clinical decline in patients with early‐stage Alzheimer’s disease (AD). The purpose of this single‐center, retrospective case series is to provide our real‐world experience with the treatment of individuals with early AD treated with lecanemab.

**Method:**

Patients with a diagnosis of early AD who were treated with lecanemab at the Infusion Center of New England in Foxboro, Massachusetts were included in this case series. Data collection included patient demographic characteristics, clinical history, lecanemab treatment exposure, and from diagnosis to treatment. Assessments included Mini‐Mental State Examination (MMSE), Montreal Cognitive Assessment (MoCA) Test for Dementia, and safety measures. Clinician perspectives from our real‐world experience with lecanemab and feedback from a patient survey were collected. Data were summarized descriptively.

**Result:**

A total of 35 patients with early AD were included in the data analysis (19 and 16 patients with 6‐month follow‐up MMSE and MoCA results, respectively; 7 patients with 12‐month follow‐up MMSE and MoCA results, respectively). Most of the patients were Caucasian (69%) and female (66%), with a mean age of 73 years. The most common diagnosis was mild AD (77%), and 63% of patients were ApoE4 carriers (46% heterozygotes; 17% homozygotes). 7 patients were initially on aducanumab and successfully transitioned to lecanemab. At 6 and 12 months, mean MMSE (baseline:23.2; 6‐months:23.6; 12‐months:24.4) and mean MoCA scores (baseline:17.4; 6‐months:19.6; 12‐months:20.6) were improved. The overall safety profile for lecanemab was similar to that observed in published clinical trials. One asymptomatic ARIA‐E (5.7%) case, 2 asymptomatic ARIA‐H (5.7%) cases, and 12 infusion‐site reactions (34%) were observed. No new long‐term safety signals were identified. 80% of survey responders reported feeling ‘very satisfied’ with lecanemab treatment and experiencing stabilization or slowing of disease progression on lecanemab.

**Conclusion:**

Findings from this case series provide real‐world evidence of the lecanemab safety and tolerability profile consistent with clinical trials which demonstrated that lecanemab slowed disease progression in early‐stage AD. To further characterize long‐term health outcomes associated with lecanemab, future studies that track patients longitudinally in a real‐world clinical setting are warranted.

